# On the Forceps and Vectis

**Published:** 1811-08

**Authors:** 


### *To the Editors of the Medical and Physical Journal.*

### *On the Forceps and Vectis.*

GENTLEMEN,

IN cases of difficult labour, when the head of the child is fixed in the cavity of the pelvis and cannot be expelled by the powers of nature, few practitioners, I believe, now make trial of the vectis, the superiority of the forceps having been so thoroughly established. Amongst the many advantages which the vectis has been said exclusively to possess, one in particular was, that the practitioner could act with it long before the head had descended low enough in the pelvis to justify him in any attempts to introduce the forceps. The advantages of the vectis in this respect is still spoken of, though I cannot perceive that the opinion is deserving of much attention. In the first place, it has been proved in Dr. Osborne's Essay, that when the head can possibly be made to pass through the pelvis, instrumental assistance can never be required *before* the head has descended sufficiently low in the cavity of the pelvis to admit of the safe and effectual application of the forceps. "This situation of the head," he observes, "is always discovered by feeling an ear of the child." In the second place, the rules in general given for the application of the vectis imply, that the head is in that situation in the pelvis which is well known to be eligible for the management of the forceps. The rule is, that "the woman may be placed on her left side in the usual posture, and then with the fore finger of the left hand *we feel for the ear of the child* next the pubis." [Burn's Midwifery]. It is quite plain, that when such rules for the application of the vectis can be observed, the situation of the head in the pelvis must be that which would constitute a *forceps case.* Whether according to Dr. Hamilton's method of applying the vectis "*over the occiput*," it is meant to use the instrument before an ear of the child can be felt, I know not; if so, here is, in Dr. Hamilton's words, a strong confirmation of Dr. Osborne's declaration, how trifling and ambiguous the effect of the vectis must be, if used before that stage of labour in which it is practicable to deliver with the forceps. The vectis being applied over the occiput, "the head," Dr. Hamilton says, "should be drawn down till the whole of it be completely within the cavity of the pelvis; to accomplish which it will, in some cases, require *the continued exertions of the practitioner for several hours.*" [See his Outlines.]

Notwithstanding

Notwithstanding the authorities in favour of the vectis, the forceps has long obtained a decided preference in general practice; so much so, that it is very questionable whether many practitioners are provided with a vectis, in case they should at any time feel disposed to commence with it those long continued exertions which Dr. Hamilton recommends. As far as my information extends on the subject, the forceps are used by nine tenths, at least, of the practitioners of the present day in the difficult labours in question. Considerable obstacles to the introduction of the forceps, however, are occasionally experienced by the most expert; and it is well known that serious misfortunes have followed the unskilful management of the instrument. It is therefore important that every circumstance that can concern the right management of the forceps should be duly considered. An unfortunate forceps case lately happened in this vicinity. The perineum was extensively lacerated, the fœces came away involuntarily, and the unfortunate patient survived only a few weeks under these calamitous circumstances.

I wish now to attend to the manner of applying the forceps. Mr. Burns, in his late publication, advises the first blade of the forceps to be passed in that direction over the head, which Dr. Denman thought best for the application of the vectis, along an "imaginary line drawn from the vertex to the chin." Mr. Burns, it should be understood, is speaking of the short forceps without convex edges. If the practitioner should prefer this kind of forceps for the purpose of applying the cup of the blades over the chin of the child; he should be aware that such a direction of the blades will not correspond with the axis of the pelvis. An imaginary line drawn from the vertex to the chin of the child, cannot in any stage of labour correspond with an imaginary line drawn from the extremity of the os coccygis to the umbilicus, which is that of the axis of the pelvis. An imaginary line continued in the direction of the forceps would, while the head lies obliquely, pass out of the *side* of the abdomen on a level, perhaps, with the umbilicus. The same imaginary line, in the direction of the forceps, would, after the face had turned into the hollow of the sacrum, be continued to one of the dorsal vertebræ.

When the forceps with convex edges is used, the cup of the blades does not rest on the chin, but upon the mastoid processes, as Dr. Osborne has observed.

Let the practitioner employ the kind of forceps he judges best (though I think the forceps with convex edges ought to be preferred.) He should, in introducing the blades, be equally attentive to the situation of the parietal protuberances, berances, as to the ears of the child; for it is of equal consequence that the blades be fixed over the one as over the other, *if the instrument is to act in corresponding lines on each side of the head.* The inaptness of the forceps to the shape of the child's head, when the blades are not fixed over the parietal protuberances, although placed in corresponding lines, not having been insisted upon, I shall beg leave to be particular in establishing the fact.

It is necessary to bear in mind, that the back part of the head begins to slope off where the parietal protuberances terminate; so that the breadth of the head at the lambdoidal suture is very considerably less than at the parietal protuberances. The space from the parietal protuberances to the lambdoidal suture, varies from an inch and a quarter to an inch and a half, or more. Now it is upon this part of the head upon which it is improper to fix *the blades* of the forceps, and a little reflection on the construction of the forceps will make the reason obvious. The forceps are adapted to act on PARALLEL SURFACES (surfaces at equal distances from each other, in the direction of the blades of the forceps); consequently, only one of the iron rims of each blade can possibly come in contact with a body of a conical shape. Now the back part of the head, from the parietal protuberances, is conical, in regard to all practical purposes, at least; hence the blades of forceps fixed here, can only press the head with the two iron rims near the parietal protuberances, the two rims near the occiput being at some distance from the head. The consequence must be that the hold of the instrument is insecure, that the head of the child suffers from the pressure being concentrated to a few points, and that the parts of the woman are in danger of injury from the edges of the blades which stand apart from the head.

As it is evident that the forceps, when placed in this situation, cannot act properly, it remains to inquire whether the directions usually given for the application of the instrument are in correspondence with the fact.

The finger is first to be passed to the ear of the child, next the pubis, as a guide to the first blade, as the ear, in this situation, is generally to be found by introducing the finger betwixt the parietal protuberance and the lambdoidal suture. "The first blade," Mr. Burns says, "follows a line drawn from the vertex to the chin, crossing the ear." If I understand the definition of the term *vertex* right, *the space where the bones slope backwards*, from the parietal protuberances, "like an obtuse angle, to the upper part of the occiput, which is a little flattened, is called the vertex." (Burns, p. 23.) "The second blade must follow a corresponding line on on the head." Both blades of the forceps, it is evident, will then be placed over the parietal bones, betwixt the protuberances and the occiput; and it is unnecessary for me again to say why it is physically impossible that the forceps can act properly in this situation. After the second blade is introduced, it is said that it may not lock easily; and a further direction is given which is not unimportant. On the presumption that the second blade, when it will not lock easily, does not follow the corresponding line, you must *adjust* it. To *adjust* is a very vague word on this occasion; however, till the blades lock easily the instrument is not *adjusted*. What happens in the attempt to rectify the direction of the second blade? The inexperienced practitioner, not having a principle to direct him, introduces the blade again, and fails. At last he has the satisfaction to succeed; and why? not because he has had the good luck to hit the corresponding line sought; no, but because he has placed the second blade on a surface, parallel to that surface upon which the first blade is laid. This parallel surface can only be found betwixt the parietal protuberance and the *coronal* suture. Let the practitioner reflect upon the anatomy of the fœtal skull, and the construction of the forceps, and he will soon find that in *practice*, if the first blade lies *behind* the one parietal protuberance, the second blade cannot antagonise it unless it be laid *before* the other.

I apprehend, however, that it is generally improper to fix the first blade of the forceps *behind* the parietal protuberance; and that the object ought to be to place the blade in a direction from the central part of the parietal protuberance to the ear. The second blade *must* then be laid on the other side of the head in a corresponding line; otherwise the blades could not act on parallel surfaces, and, of course, could not possibly act properly. In introducing the second blade then, the situation of the parallel protuberance must be strictly attended to; for should the blade be fixed on the parietal bone too far back, there would be a considerable hiatus betwixt the posterior iron rim of the blade and the head. The inflexible bodies, therefore, which encompass the back part of the head, would require much additional space; and a proportionally under part of the arch of the pubis to pass under. The *perineum*, therefore, being very much on the stretch, would, in addition to the evils already stated, be in great danger of laceration.

I am, Gentlemen,

Your obedient Servant,

A. B. G.

*May* 13, 1811.

